# Current patient attitudes to artificial intelligence applications in radiology

**DOI:** 10.1093/bjr/tqag077

**Published:** 2026-04-02

**Authors:** Rory H Maclean, Iqbal Aniq, Jack Delaney, Yoyel Kang, Robert O’Shea, Asif Mazumder, Carolyn Horst, Vicky Goh

**Affiliations:** Department of Cancer Imaging, School of Biomedical Engineering and Imaging Sciences, King’s College London, London WC2R 2LS, United Kingdom; Radiology Department, Guy’s and St. Thomas’ NHS Foundation Trust, London SE1 7EH, United Kingdom; Radiology Department, Guy’s and St. Thomas’ NHS Foundation Trust, London SE1 7EH, United Kingdom; Radiology Department, Guy’s and St. Thomas’ NHS Foundation Trust, London SE1 7EH, United Kingdom; Department of Cancer Imaging, School of Biomedical Engineering and Imaging Sciences, King’s College London, London WC2R 2LS, United Kingdom; Radiology Department, Guy’s and St. Thomas’ NHS Foundation Trust, London SE1 7EH, United Kingdom; Department of Cancer Imaging, School of Biomedical Engineering and Imaging Sciences, King’s College London, London WC2R 2LS, United Kingdom; Department of Cancer Imaging, School of Biomedical Engineering and Imaging Sciences, King’s College London, London WC2R 2LS, United Kingdom

**Keywords:** patient participation, artificial intelligence, prospective studies, delivery of health care, surveys and questionnaires

## Abstract

**Objectives:**

Healthcare systems are now funding implementation of artificial intelligence (AI) algorithms in radiology, which will change the experience of care for patients. Currently, there is still limited evidence of patient attitudes to AI implementation in healthcare. We aimed to determine current attitudes to AI of people attending hospital for diagnostic imaging.

**Methods:**

This prospective study was conducted at a tertiary hospital network. Following ethical approval and informed consent, an 18-item questionnaire was administered to patients attending for outpatient imaging, assessing their views on AI. Factor analysis was undertaken to identify themes.

**Results:**

In total, 162 people completed the questionnaire; 56% of whom were female (91/162). Most people thought that AI in healthcare would be useful (78%) and should be used (64%). People felt strongly that doctors should be responsible for decisions involving AI (71%). Three latent factors were identified: “utility and safety,” “interaction,” and “comparability to doctors.” People were positive about the utility and safety of AI, were concerned about a loss of personal interaction, and compared AI unfavorably to doctors. There was strong opposition to autonomous AI decisions.

**Conclusions:**

The findings of this study map current patient acceptability of AI in healthcare and should inform strategies to balance AI ethical implementation with delivering value to patients and the healthcare system.

**Advances in knowledge:**

This study provides insights into current patient attitudes to AI in healthcare in a UK setting where AI tools are actively being deployed, building on prior European surveys and guiding ongoing AI design and implementation.

## Introduction

In recent years there has been an increase in both the number of AI medical devices on the market and public discourse around AI technology—the latter largely driven by the emergence of large language models and generative AI. There is renewed ambition to adopt healthcare AI driven by the need to address workforce challenges and increase productivity, as evidenced in the United Kingdom where the government has committed £21million to implementing diagnostic AI tools in radiology. Despite this, there remain considerable barriers to adoption of AI tools in radiology, including concerns around data robustness and data privacy,^1^ misalignment with clinical workflows,^2^ lack of strategic management resources and the need for changes in professional roles.^3^

To earn public acceptance of AI in healthcare, the healthcare system must understand the current state of public trust in the technology, and determine the “facilitating conditions” necessary,^4^ including safety measures, regulatory frameworks, legal responsibilities, and clinical operating procedures. Prior studies of public attitudes to healthcare AI in Europe have found limited enthusiasm for diagnostic AI and a preference for human interaction.^5^ Trust in AI is higher among those who believe in its efficiency, and lower in those who prioritize personal interactions with clinicians.^6^

With the growing public acceptance of AI technology in other domains, we lack a view of current public attitudes to AI in healthcare. To build understanding, we developed a questionnaire to assess the attitudes of people attending hospital for diagnostic imaging. The results of this study should inform efforts to implement healthcare AI and enable future re-assessment of public attitudes to AI.

## Methods

This was a prospective study that was ethically approved by the Health Research Authority (22/SC/0338). All participants gave written informed consent after reviewing the participant information sheet. Patient and public involvement groups in the hospital network were involved in development of the study.

### Participants

A convenience sample of participants attending for radiological examinations at a tertiary referral hospital network between September 2, 2023 and January 4, 2024 were approached. Inpatients and those undergoing urgent imaging tests were excluded to minimize the burden of research participation on acutely unwell patients. This convenience sample was intended to represent typical patients attending for an investigation at a radiology department.

### Attitudes to AI questionnaire

A questionnaire was developed to determine attitudes to AI in healthcare, informed by previous studies developing a 48-item attitudes to AI questionnaire in a Dutch population.^5^^,^^7^ In their questionnaire, Ongena et al^5^ found 5 latent factors: “distrust and accountability” (15 items), “procedural knowledge” (8 items), “personal interaction” (7 items), “efficiency” (5 items), and “being informed” (4 items). This study’s questionnaire used similar questions and themes to that of Ongena et al,^5^ with the aim of being fast to administer, straightforward to complete, and relevant to patients. As such, it was decided to use a smaller number of question items (18) and to use item-specific 5-level Likert responses, which have a lower cognitive burden than agree-disagree responses.^8^ The question items in this study were hypothesized to represent latent factors “utility,” “safety,” “comparability to doctors,” and “patient interaction with AI.” Demographic information including age, self-reported gender, self-reported ethnicity, and first part of postal code were also collected. The questionnaire was self-administered taking around 15 minutes to complete. Questionnaire data were anonymized before analysis.

### Statistical analysis

Statistical power was calculated using simulation (*n* = 1000 simulations), for both Kendall correlation and comparison of 2 groups using the Wilcoxon rank-sum test. For Kendall correlation, at target correlation 0.3 and alpha = .05, a sample size of 100 yielded a power of 0.83. For comparison of 2 groups using the Wilcoxon rank-sum test, at effect size 0.5 (difference in means) and alpha = .05, a total sample size of 140 yielded a power of 0.82.

Data missingness was assessed, and missing question item values were imputed with the median; missing demographic data were excluded from the corresponding analysis. Five-level Likert responses were normalized to the same direction: 5 is supportive of AI and 1 opposes AI, to improve comparison between question items.

Correlation between questions was assessed using the “Kendall” method for non-normal distributions. Exploratory factor analysis (rotation: “oblimin,” method: “minres”) was performed to determine latent factors. Parallel analysis (100 iterations) and inspection of the Scree plot was used to determine the optimal number of factors. Where a question item cross-loaded onto 2 factors, the factor with the larger eigenvalue was chosen. Factors were named by consensus interpretation of the question items, considering the eigenvalue weights. Scores were calculated for each factor by addition of item values.

Factor scores were compared across demographic groups both visually with distribution plot and statistically using pairwise 2-sided Wilcoxon Rank Sum Tests with “Holm” correlation for multiple comparisons.^9^ Ordinal principal components analysis (Gifi package) was used to assess the relationship between factor scores, independent question items, and demographic factors. Statistical computations were performed using R version 4.3.0.^10^

## Results

### Participants

In total, 162 participants completed the questionnaire; 56% (91/162) were female, 43% (70/162) were male, and 1 person did not declare (Table 1); 62% (100/162) identified as “White,” 19% (30/162) “Black, African, Caribbean or Black British,” and 10% (16/162) “Asian or Asian British.” The modal age groups were “30 to 49” (57/162, 35.4%) and “50 to 69” years old (57/162, 35.4%). Ethnicity and gender data were complete; data missing for age was 0.61% (1/162). The average data missing of question items was 0.38% (11/2916) with no more than 1 missing question item per participant. Most participants lived in an urban area (South London) and the remainder in the surrounding region (other areas of Greater London), with postcode information indicating a breadth of socioeconomic backgrounds.

**Table 1:** The study cohort.

	*N* (%)
Total	162
Age	
18-29	27 (16.8)
30-49	57 (35.4)
50-69	57 (35.4)
70-89	18 (11.2)
90+	2 (1.2)
Gender	
Female	91 (56.2)
Male	70 (43.2)
Prefer not to say	1 (0.6)
Ethnicity	
Asian or Asian British	16 (9.9)
Black, African, Caribbean, or Black British	30 (18.5)
Mixed or multiple ethnic groups	3 (1.9)
Other ethnic group	9 (5.6)
Persian	1 (0.6)
Prefer not to say	3 (1.9)
White	100 (61.7)

### Attitudes to AI in healthcare

**Figure 1:**
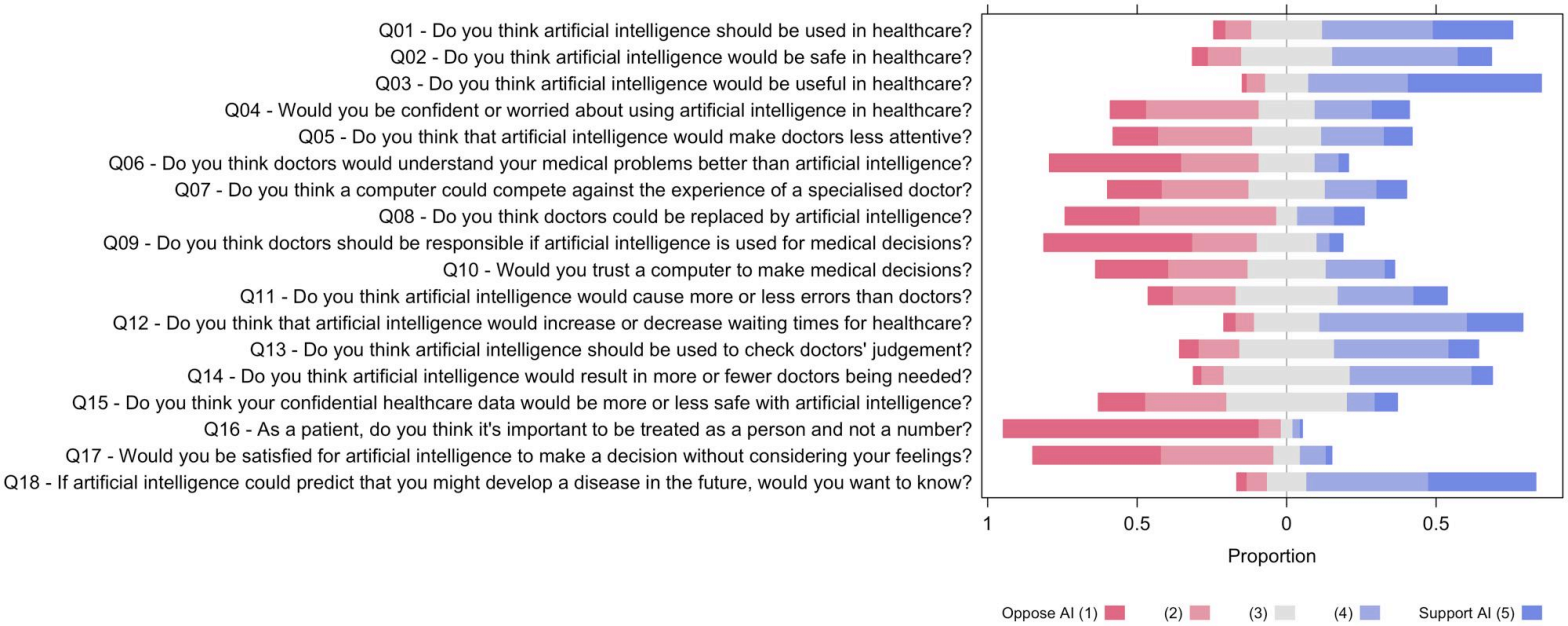
Level of support for the 18 Attitudes to AI questions (Likert plot).

People responded to 18 attitudes to AI in healthcare questions with 5-level Likert responses, oriented such that 1 opposed, 3 was ambivalent, and 5 favored AI (Figure 1). Most people thought that AI would be useful (Q03: 78% favored vs. 7% opposed; the most favored question item) and should be used in healthcare (Q01: 64% favored vs. 12% opposed). At the healthcare system level, a large majority thought that AI would decrease waiting times (Q12: 68% favored vs. 10% opposed). A large majority would want to know about an AI prediction of future disease (Q18: 77% favored vs. 10% opposed). Whilst most people thought that AI would be safe in healthcare (Q02: 53% favored vs. 16% opposed), they also thought that you could not trust a computer to make medical decision (Q10: 50% distrusted AI vs. 23% trusted AI). Many thought that confidential health data would be less safe with AI (Q15: 43% vs. 15%). It was strongly felt that a computer cannot compete against a specialist doctor (Q07: 47% vs. 27%), however there was ambivalence about whether AI would cause more or less errors than doctors (Q11: 36% favored AI, 35% ambivalent, 29% opposed AI). People thought that AI should be used to check doctors’ judgment (Q13: 48% vs. 20%) and that the doctor should be responsible for decisions involving AI (Q09: 71% vs. 9%). People felt very strongly that it is important to be treated as a person (Q16: 93% vs. 3%) and would not be satisfied with an AI decision that did not consider their feelings (Q17: 80% vs. 11%). The question of whether doctors could be replaced by AI (Q08) was polarizing, with only 7% ambivalent, 22% in agreement, and 70% thinking otherwise. Correlation analysis of 18 question items revealed positive correlation between many question items (Supplementary Material), supporting the existence of latent factors.

### Factor analysis revealed 3 latent factors

Factor analysis revealed 3 latent factors, which were associated with 16 of the 18 question items. For clarity, we have called these 3 factors: Factor 1 (F1) “utility and safety,” Factor 2 (F2) “interaction” and Factor 3 (F3) “comparability to doctors” (Table 2). F1 was most strongly associated with Q01 “Do you think artificial intelligence should be used in healthcare?,” and Q02 “Do you think artificial intelligence would be safe in healthcare?” F2 was most strongly associated with Q16 “As a patient, do you think it’s important to be treated as a person and not a number?” and Q17 “Would you be satisfied for artificial intelligence to make a decision without considering your feelings?” F3 was most strongly associated with Q08 “Do you think doctors could be replaced by artificial intelligence?” and Q07 “Do you think a computer could compete against the experience of a specialized doctor?” Two questions were independent of the 3 factors: Q09 “Do you think doctors should be responsible if artificial intelligence is used for medical decisions?” (characterized as “autonomous”) and Q14 “Do you think artificial intelligence would result in more or fewer doctors being needed?”

**Table 2:** Factor analysis of 18 question items revealed 3 latent factors and 2 independent questions.

Question	F1: utility + safety	F2: interaction	F3: comparability
Q01: Do you think artificial intelligence should be used in healthcare?	0.85	0.00	0.00
Q02: Do you think artificial intelligence would be safe in healthcare?	0.83	0.00	0.00
Q03: Do you think artificial intelligence would be useful in healthcare?	0.83	0.00	0.00
Q04: Would you be confident or worried about using artificial intelligence in healthcare?	0.72	0.00	0.00
Q05: Do you think that artificial intelligence would make doctors less attentive?	0.59	0.00	0.00
Q06: Do you think doctors would understand your medical problems better than artificial intelligence?	0.00	0.31	0.00
Q07: Do you think a computer could compete against the experience of a specialized doctor?	0.00	0.00	0.61
Q08: Do you think doctors could be replaced by artificial intelligence?	0.00	0.00	0.71
Q09: Do you think doctors should be responsible if artificial intelligence is used for medical decisions?	0.00	0.00	0.00
Q10: Would you trust a computer to make medical decisions?	0.49	0.34	0.00
Q11: Do you think artificial intelligence would cause more or less errors than doctors?	0.62	0.00	0.00
Q12: Do you think that artificial intelligence would increase or decrease waiting times for healthcare?	0.51	0.00	0.00
Q13: Do you think artificial intelligence should be used to check doctors’ judgement?	0.46	0.00	0.00
Q14: Do you think artificial intelligence would result in more or fewer doctors being needed?	0.00	0.00	0.00
Q15: Do you think your confidential healthcare data would be more or less safe with artificial intelligence?	0.00	0.00	0.36
Q16: As a patient, do you think it’s important to be treated as a person and not a number?	0.00	0.77	0.00
Q17: Would you be satisfied for artificial intelligence to make a decision without considering your feelings?	0.00	0.54	0.00
Q18: If artificial intelligence could predict that you might develop a disease in the future, would you want to know?	0.46	0.00	0.00

### Attitudes to AI differed between factors

**Figure 2:**
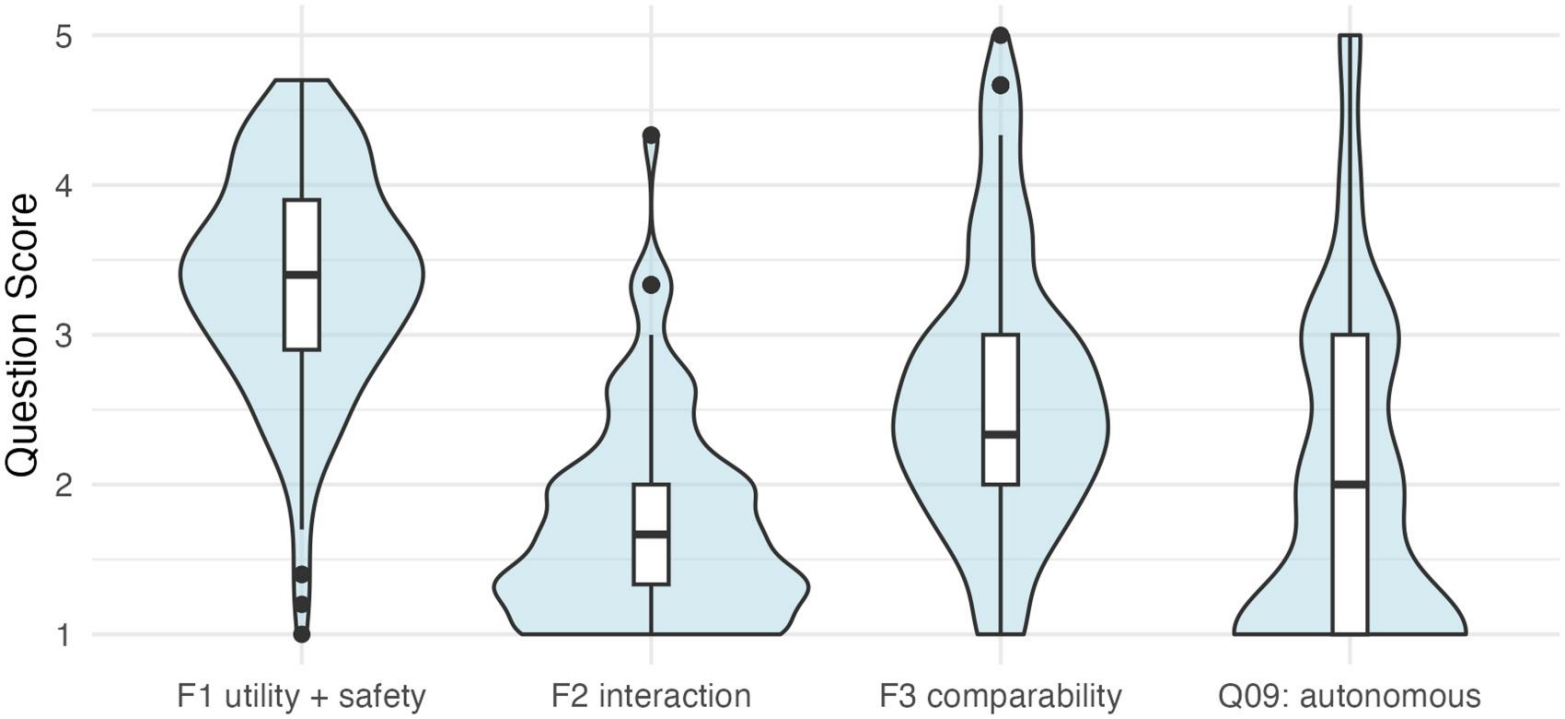
Attitudes to AI differed between factors. F2 interaction vs. F1 utility + safety: *P ≤* .001, F3 comparability vs. F1 utility + safety: *P ≤* .001, F3 comparability vs. F2 interaction: *P ≤* .001, Q09: autonomous vs. F1 utility + safety: *P ≤* .001, Q09: autonomous vs. F3 comparability: *P ≤* .001, Q09: autonomous vs. F2 interaction: *P* = 1.00.

Factor scores were calculated for the 3 latent factors, on the original scale from 1: “oppose AI” to 5: “favor AI.” There were significant pairwise differences in support for the factors (*P < .*001 for all comparisons, except Q09 vs. F2) (Figure 2, Supplementary Material). Participants opposed “interaction” with AI instead of doctors (F2: mean 1.7, SD 0.7), but there was strong support for the “utility and safety” of AI (F1: mean 3.4, SD 0.7). AI was felt to compare unfavorably with doctors (F3: mean 2.6, SD 0.9), and most were opposed to autonomous AI use, responding that doctors should be responsible for AI decisions (Q09: mean 1.9, SD 1.1).

### Attitudes to AI differed by gender but not by age or ethnicity

**Figure 3:**
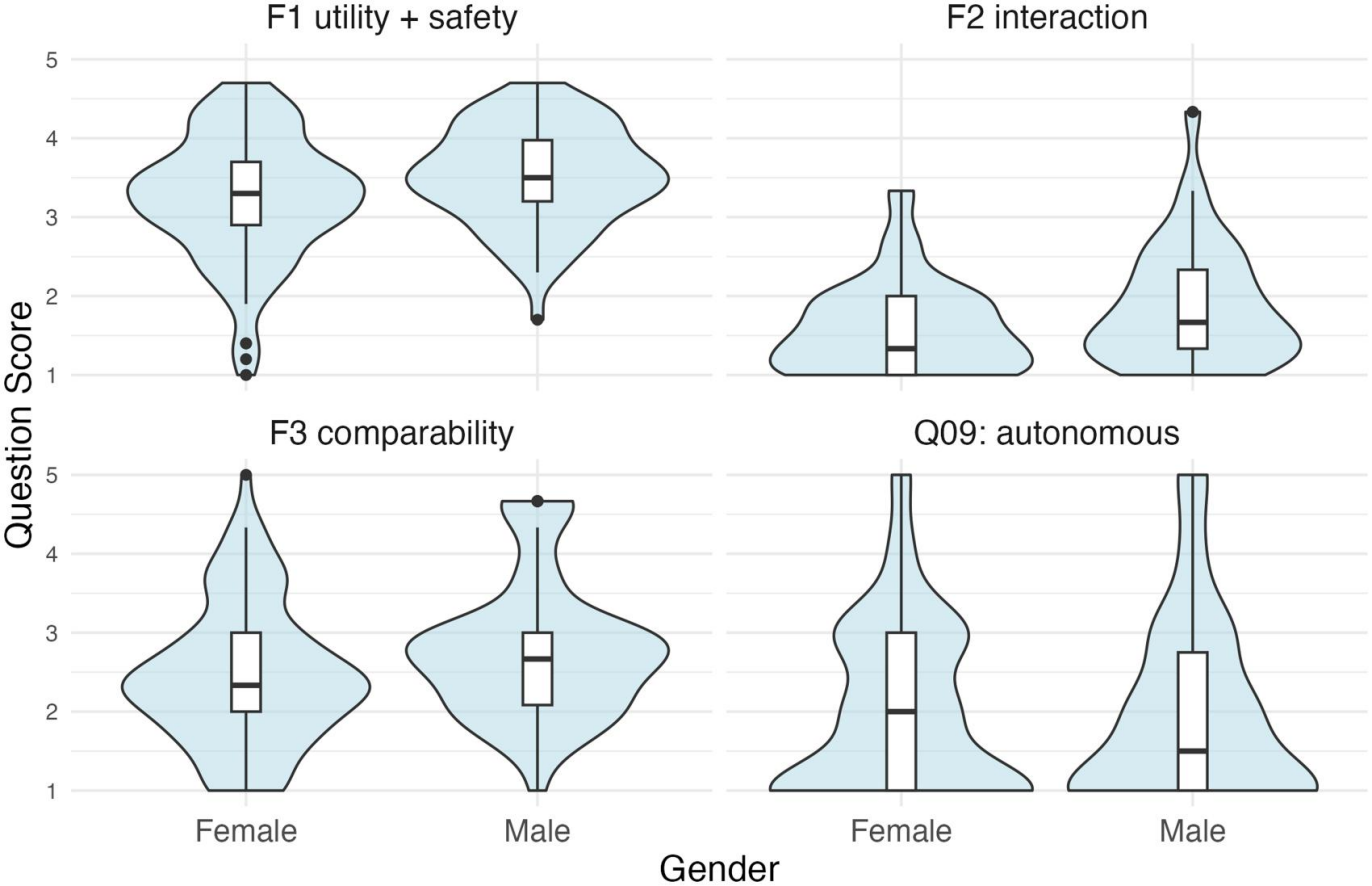
Attitudes to AI differed by gender. F1 utility + safety: *P = *.088; F2 interaction: *P = *.037; F3 comparability: *P = *.018; Q09: autonomous: *P = *.525.

Female respondents were more likely to oppose interaction with AI (*P = .*037), favor doctors to AI (*P = .*018), and did not support the utility and safety of AI to the same extent (*P = .*088) (Figure 3). Support for autonomous AI did not differ by gender (*P = .*525). There was no evidence that attitudes to AI differed by age or ethnicity (Supplementary Material).

## Discussion

Our study examined the attitudes to AI in healthcare of participants attending a hospital radiology department for an examination, using a questionnaire designed to be straightforward to complete, and relevant to patients. The study population was representative of the general population in our urban area in terms of age, gender, and ethnicity. The responses to 18 questions revealed 3 key factors shaping attitudes: “utility and safety,” “interaction,” and “comparability to doctors.” Respondents generally supported the utility and safety of AI but were opposed to interactions with an AI system in place of a doctor and viewed doctors as superior to AI systems. There was strong opposition to AI making autonomous decisions. Females vs. males were more likely to have concerns about AI on the interaction, comparability, and utility and safety scales.

Our study’s questionnaire was informed by Ongena et al,^5^ allowing comparisons across the studies. The 2 studies shared similar factors: “distrust and accountability” (Ongena et al^5^) and “utility and safety” (this study); “personal interaction” and “interaction”; “efficiency” and “comparability to doctors.” Overall, the attitudes to AI were different between the 2 populations. In our study, the public expected AI to be useful and safe, in contrast to Ongena et al’s^5^ finding of a generally negative attitude to trust and accountability. The difference between the 2019 Dutch study and our UK study may be related to a systematic difference in attitudes between the cultural contexts, or a shift in attitudes with the passing of time and increasing AI use in other domains.

However, both studies agreed that people value personal interaction and a human dimension to healthcare, a finding supported by a systematic review of patient attitudes.^11^ In our study the public remain concerned that AI in healthcare might lead to weakening of the clinician-patient relationship. This is also reflected in a 2024 online UK survey by the Health Foundation that found that the UK public and healthcare professionals expect AI to improve care but have concerns about the impact on the human side of care.^12^ The development and implementation of AI systems must safeguard and enrich the personal interaction between patient and doctor.

In our study public attitudes to utility and safety were aligned. The key factor may be underlying “belief in AI.” In the AI field, “capability” and “safety” are distinct goals. Despite high overall performance, AI systems may struggle with rare cases, not often represented in the training data.^13^ For diagnostic AI, utility and safety are closely related via diagnostic performance. However, an AI system with high diagnostic performance could also be poorly implemented, leading to safety issues such as automation bias, overfitting to clinical pathways, and cybersecurity vulnerabilities. Further work is needed to understand the priorities of the public regarding specific utility and safety issues.

Our study confirmed there is ongoing public concern about autonomous AI decisions. For example, within radiology, the CE-marked “ChestLink” AI system can autonomously report normal chest X-ray studies. Public acceptability will be dependent on the setting in which AI is used, clinical risks, and patient experience, and certain use cases are likely to be acceptable.^14^ The previously sharp distinction between autonomous and computer-aided decision-making is increasingly blurred. Recent work has extended operation mode “human in the loop,” ie, with human involvement in each decision, to “human on the loop,” ie, without human involvement in each decision but instead with continuous monitoring.^15^ We must work to better understand public acceptability across use cases and degrees of automation.

Whilst all autonomous use cases were opposed, Thornton et al^12^ found that the public were in favor of AI assistance in the same use cases if supervised by a human, particularly for administrative tasks, but less so for decisions on treatment.

There were limitations to our study. This study was conducted in an urban hospital network, and generalizability to non-urban settings is unknown. The number of questionnaire items was limited and was not designed to be fully inclusive of all technical aspects, to improve participation and minimize incomplete questionnaires. Thus, this survey provides a general overview of attitudes rather than an in-depth exploration, which would be better addressed with a qualitative study design.

In conclusion, our study has mapped current patient acceptability of AI in healthcare, and should inform the ongoing implementation of AI in radiology. While there appears to be greater acceptance of the use of AI technology in healthcare, there is still strong opposition to autonomous AI and any weakening of the clinician-patient relationship. Work to understand public acceptance of AI in healthcare across different autonomy levels is important for the ethical implementation of AI and to deliver value to patients. This questionnaire can be used to regularly evaluate patients’ attitudes to AI in the future, as AI implementation further shapes and changes the public discourse.

## Supplementary Material

tqag077_Supplementary_Data
