# Supplementary material for: Current patient attitudes to artificial intelligence applications in radiology
Source: Br J Radiol. 2026 Apr 2;99(1182):1149–55. doi: 10.1093/bjr/tqag077 (PMC13195512; doi:10.1093/bjr/tqag077)

# Supplemental material

## Figure S1. Kendall correlation analysis of the attitudes to AI 18-item questionnaire. The colour legend represents the correlation coefficient.


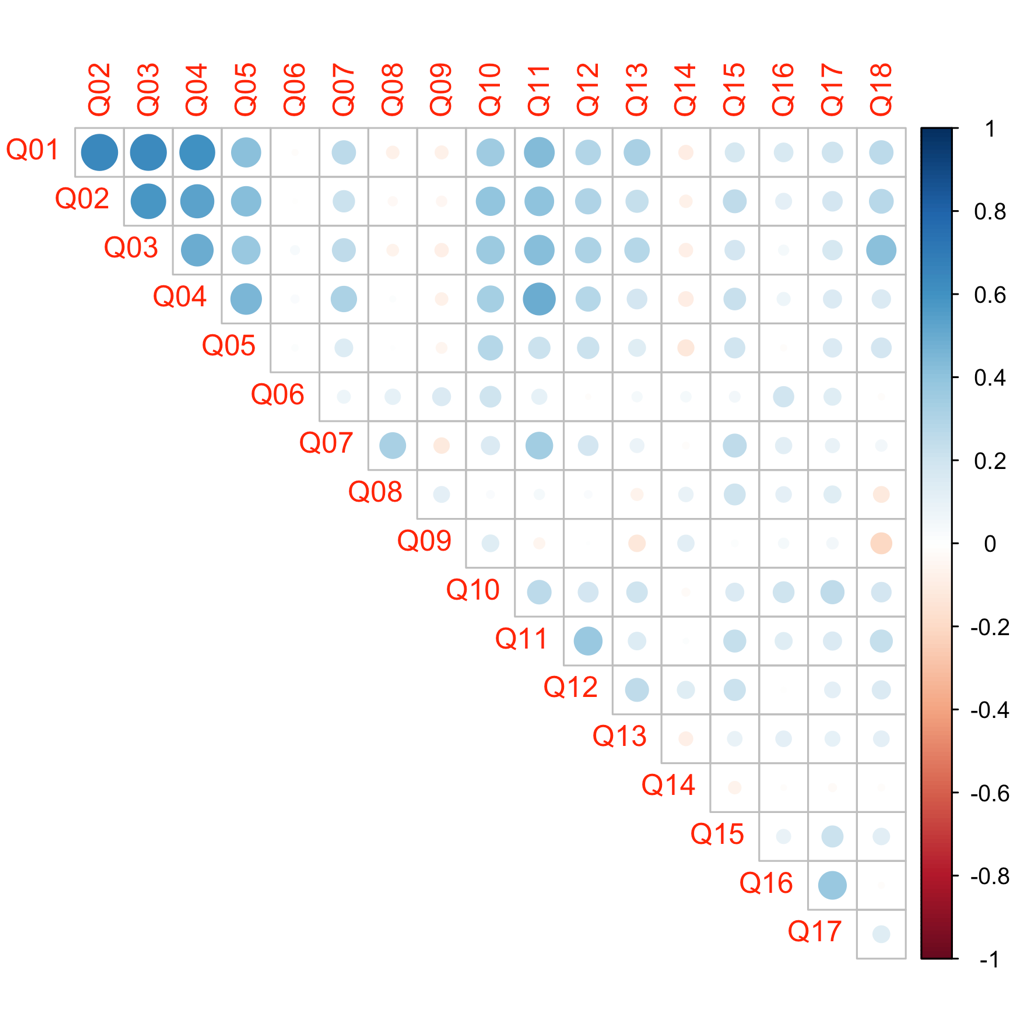


## Figure S2. Ordinal Principal Components Analysis demonstrated relationships between the factors and demographics. The component variable loadings for two principal components are plotted for the factors (F1-3), Q09: autonomous, age, and gender.

##
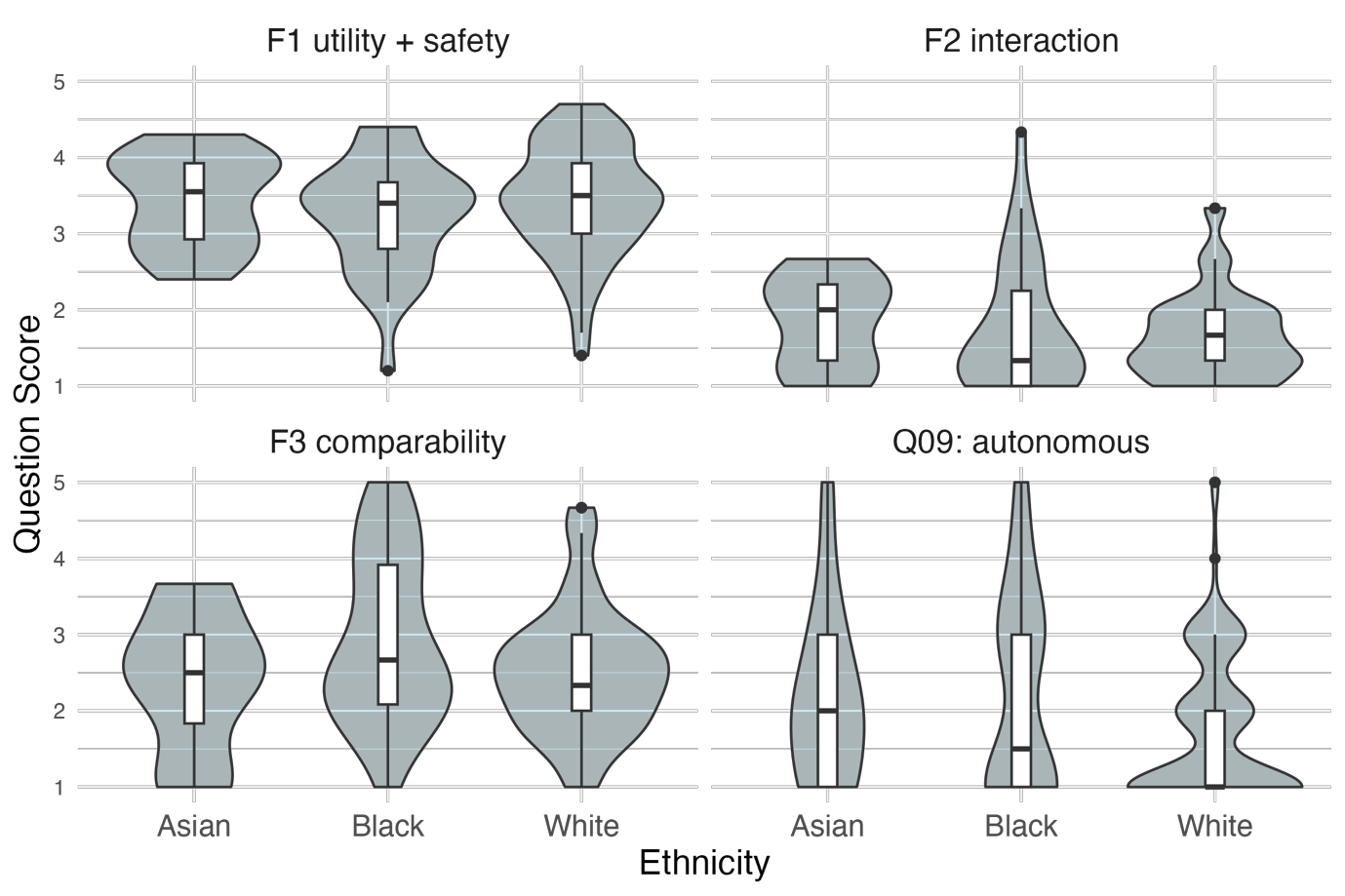
Figure S3. There was no evidence that attitudes to AI differed between age groups. The average question score for the three factors (F1-3) and Q09: autonomous are shown by ethnicity group.

## Figure S4. There was no evidence that attitudes to AI differed between age groups. Average question score for three factors (F1-3) and Q09: autonomous are shown by age group.


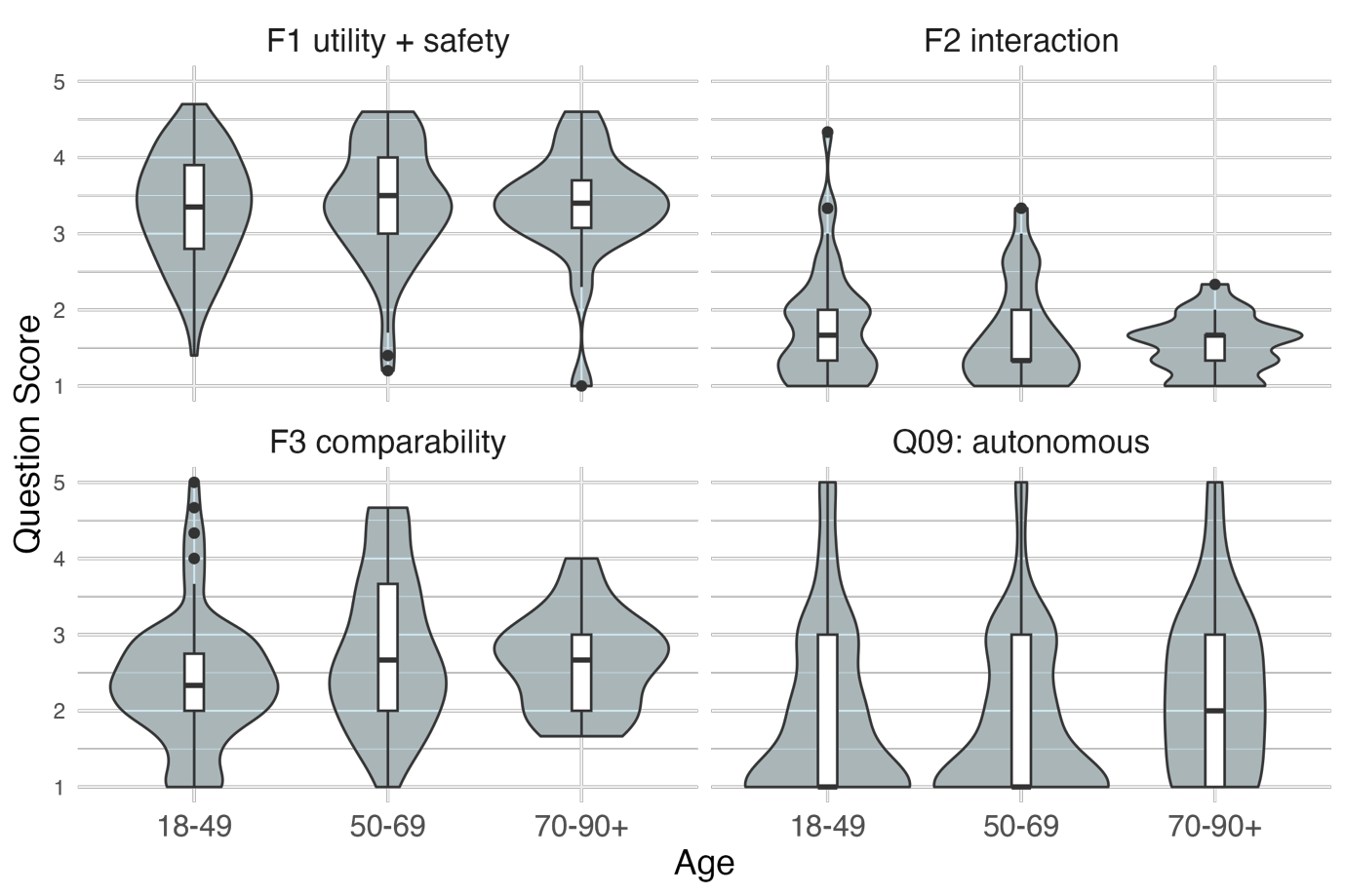

Supplement: tqag077_Supplementary_Data [file tqag077_supplementary_data.zip › Supplemental material.docx]
