# Supplementary figures and images for: Current patient attitudes to artificial intelligence applications in radiology
Source: Br J Radiol. 2026 Apr 2;99(1182):1149–55. doi: 10.1093/bjr/tqag077 (PMC13195512; doi:10.1093/bjr/tqag077)

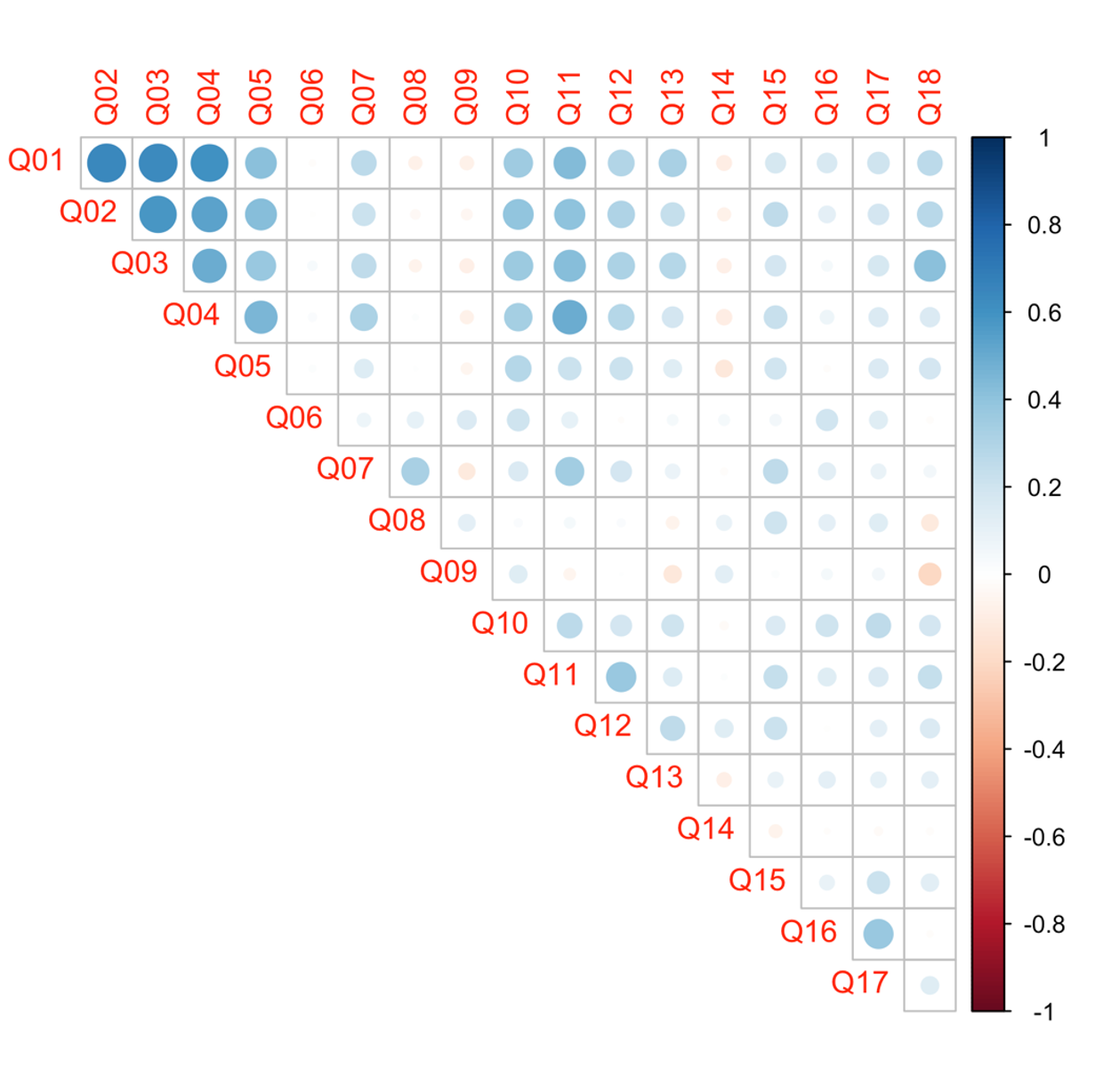

Supplement: tqag077_Supplementary_Data [file tqag077_supplementary_data.zip › supp_fig_1.png]

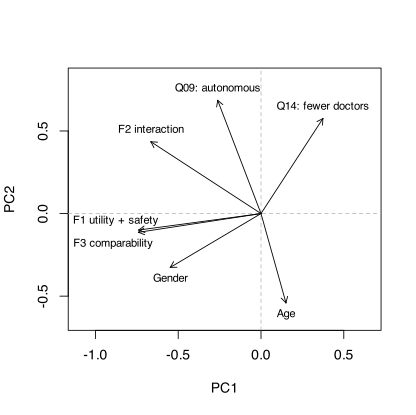

Supplement: tqag077_Supplementary_Data [file tqag077_supplementary_data.zip › supp_fig_2.png]

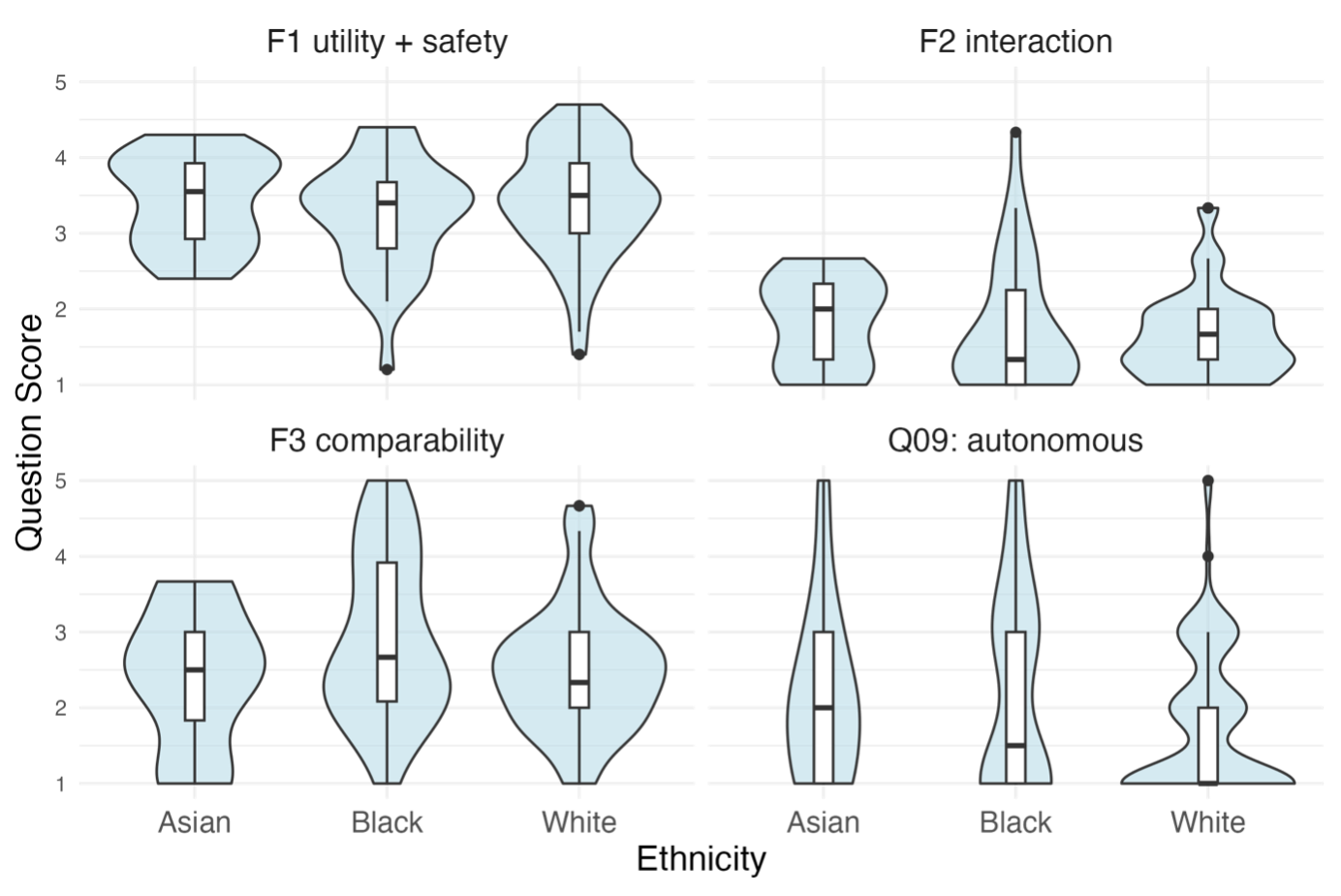

Supplement: tqag077_Supplementary_Data [file tqag077_supplementary_data.zip › supp_fig_3.png]

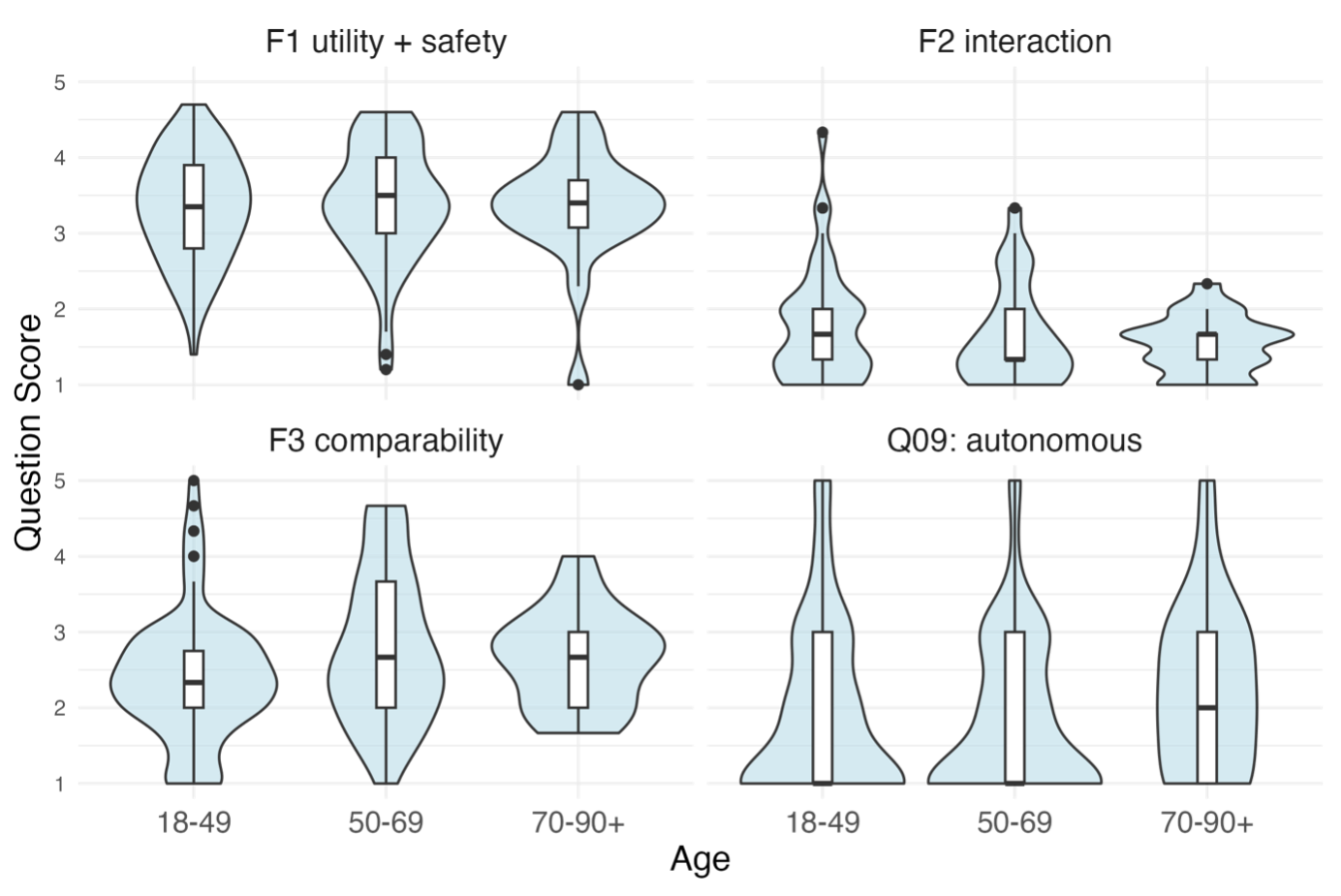

Supplement: tqag077_Supplementary_Data [file tqag077_supplementary_data.zip › supp_fig_4.png]
